# Predictive model estimating the decrease of postoperative gastrointestinal quality of life index (GIQLI) in patients after elective laparoscopic sigmoid resection for diverticular disease

**DOI:** 10.1007/s00423-021-02186-w

**Published:** 2021-05-25

**Authors:** Alberto Posabella, Daniel Christian Steinemann, Raoul André Droeser, Nadshathra Varathan, Selin Göksu Ayçiçek, Fabio Nocera, Markus von Flüe, Niccolò Rotigliano, Ida Füglistaler

**Affiliations:** 1 Clarunis, Department of Visceral Surgery, University Centre for Gastrointestinal and Liver Disease, St Clara Hospital and University Hospital, Kleinriehenstrasse 30, 4058 Basel, Switzerland; 2 Department of Surgery, University Hospital Basel, Spitalstrasse 23, 4031 Basel, Switzerland

**Keywords:** Laparoscopic sigmoid resection, Gastrointestinal quality of life, GIQLI, Bowel function, Linear regression model

## Abstract

**Background:**

Growing consideration in quality of life (QoL) has changed the therapeutic strategy in patients suffering from diverticular disease. Patients’ well-being plays a crucial role in the decision-making process. However, there is a paucity of studies investigating patients’ or surgery-related factors influencing the postoperative gastrointestinal function. The aim of this study was to investigate in a predictive model patients or surgical variables that allow better estimation of the postoperative gastrointestinal QoL.

**Methods:**

This observational study retrospectively analyzed patients undergoing elective laparoscopic sigmoidectomy for diverticulitis between 2004 and 2017. The one-time postoperative QoL was assessed with the gastrointestinal quality of life index (GIQLI) in 2019. A linear regression model with stepwise selection has been applied to all patients and surgery-related variables.

**Results:**

Two hundred seventy-two patients with a mean age of 62.30 ± 9.74 years showed a mean GIQLI of 116.39±18.25 at a mean follow-up time of 90.4±33.65 months. Women (*n*=168) reported a lower GIQLI compared to male (*n*=104; 112.85±18.79 vs 122.11±15.81, *p*<0.001). Patients with pre-operative cardiovascular disease (*n*=17) had a worse GIQLI (106.65 ±22.58 vs 117.08±17.66, *p*=0.010). Finally, patients operated less than 5 years ago (*n*=63) showed a worse GIQLI compared to patients operated more than 5 years ago (*n*=209; 111.98±19.65 vs 117.71±17.63, *p*=0.014).

**Conclusions:**

Female gender and the presence of pre-operative cardiovascular disease are predictive for a decreased postoperative gastrointestinal QoL. Furthermore, patients’ estimation of gastrointestinal functioning seems to improve up to 5 years after surgery.

**Graphical abstract:**

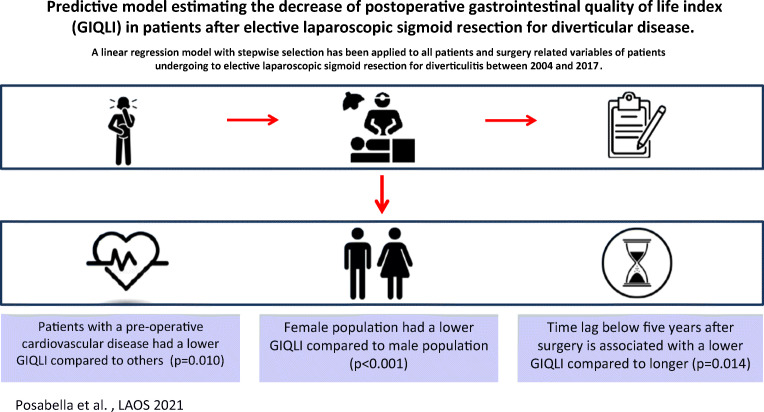

**Supplementary Information:**

The online version contains supplementary material available at 10.1007/s00423-021-02186-w.

## Introduction

During the last decades, there is a growing attention to the quality of life (QoL) of patients after abdominal surgery, particularly in those suffering from diverticular disease [1, 2]. Recently the guidelines on the treatment of diverticulitis have changed in particular concerning the role of the surgery. If previously the number of diverticulitis episodes represented one of the main criteria to decide for sigmoidectomy, nowadays the patient’s well-being and more generally their QoL play a crucial role in that decision-making process [2–4]. Hence, the treatment of uncomplicated diverticulitis has evolved to a tailored approach, and a major consideration has been given to the evaluation of their gastrointestinal symptoms [3–5].

Confirmed by recent literature, patients undergoing elective laparoscopic sigmoid resection for diverticulitis report an improvement in their gastrointestinal symptoms compared to patients treated conservatively [6–8]. Unfortunately, previous studies did not assess in detail patients’ related demographic data (as age, sex, pre-operative comorbidities, indication for surgical treatment), intraoperative variables (such as kind of anastomosis, vascular approach to the inferior mesenteric artery, conversion to laparotomy, use of drainage), and postoperative morbidity on gastrointestinal QoL.

Thus, the purpose of the current study was to evaluate the gastrointestinal QoL after elective laparoscopic sigmoid resection using the gastrointestinal quality of life index (GIQLI). The GIQLI is a 36-item gastrointestinal-specific questionnaire designed to assess, in clinical practice, the gastrointestinal function of patients [9]. Although focusing on the core gastrointestinal symptoms, four other different subdomains (physical, psychological, social, and disease-specific items) widely investigate different aspects of the QoL of the patients [9]. All different subdomains explored by the GIQLI were assessed as well as potential risk factors for a decreased postoperative GIQLI. Our attention was focused to elucidate any predictive role of the variables analyzed.

## Methods

### Study design

Data were retrospectively collected from patients undergoing elective laparoscopic sigmoid resection for diverticular disease between 2004 and 2017 at the St. Clara Hospital in Basel, Switzerland.

Patients undergoing an emergency or a primary open resection were excluded. Data collected comprise patient’s demographic, pre-operative comorbidities, intraoperative surgical technique, postoperative morbidity, and mortality at 30 days.

Thus, in 2019 the 36-item gastrointestinal quality of life index (GIQLI) was sent by mail to all eligible patients to collect their postoperative outcomes according to the Table S1 (see supporting information) [9]. Along with the questionnaire, a patient information letter explaining the purpose of the study and a written informed consent were enclosed. Patients who did not return the survey despite our reminder phone call, or those who did not agree to participate, or those who deceased prior to the time of the assessment were excluded from the study.

Patients who successfully completed and returned the questionnaire were also contacted by telephone by the same investigator to assess actual comorbidities and possible subsequent abdominal surgery in the time frame between sigmoid surgery and survey. In particular, patients under regular medication for gastrointestinal tract diseases as well as patients who underwent surgery on the upper or lower GI tract affecting the intestinal function were excluded from the final analysis.

Finally, a linear regression model with stepwise selection has been applied to all data analyzed from this population in order to find the best predictive combination of variables to estimate the postoperative GIQLI.

The study was conducted in compliance with the current version of the Declaration of Helsinki and was approved by the ethics committee of the Northwestern and Central Switzerland (EKNZ 2018-00318).

### Surgical technique

To rule out malignancy, all patients had a pre-operative colonoscopy at least 2 weeks before the surgical procedure. The day before the intervention, mechanical bowel preparation and a thrombotic prophylaxis was performed. By induction of general anesthesia, antibiotic prophylaxis was given and repeated, if necessary, every 4 h (metronidazole 500 mg iv and cefuroxime 2 g iv). Once the CO2 pneumoperitoneum was established, the laparoscopic procedure began with the dissection of the gastrocolic ligament to reach a complete mobilization of the splenic flexure. According to the twelve involved surgeon’s preferences, the vascular approach to the IMA was distinguished between central or peripheral ligation. In the first case, the inferior mesenteric vein was firstly identified at the inferior pancreatic margin close to the Treitz ligament and sectioned between clips. The IMA was detected at its origin from the aorta (“high tie”) and transected with a vascular stapler (Endo GIA™ 30/45-mm Articulating Vascular/Medium Reload with TriStaple™ Technology, Covidien) after routine identification and preservation of the autonomic nerves of the superior hypogastric plexus.

On the contrary, in the peripheral ligation of the IMA, the mesentery dissection was performed close to the colonic wall sparing the left colic artery as well as the superior rectal artery.

Finally, the colon was then transected with a linear stapler (Endo GIA™ 45/60-mm Articulating Medium/Thick Reload with Tri-Staple™ Technology, Covidien), and the colorectal anastomosis, when applicable, was performed trans-anally in a double stapling technique. The side-to-end anastomosis was considered the first choice, while the side-to-side and the end-to-end anastomosis were only performed in particular intraoperative conditions (e.g., lack of adequate length for anastomosis). The sigmoid specimen was retracted through a Pfannenstiel incision or enlargement of the left lower abdominal incision.

### Outcome measurements

The one-time postoperative QoL was assessed with the gastrointestinal quality of life index (GIQLI) [9]. This is a validated gastrointestinal QoL questionnaire consisting of 36 questions investigating the core gastrointestinal symptoms as well as physical, psychological, social, and disease-specific issues. Each question has a score ranking from 0 (worst) to 4 (best). The maximal obtainable score is 144, reflecting an optimal QoL without any symptoms, as described in Table S1 (see supporting information).

With the aim to assess the later developed comorbidities, we conducted a telephone survey using the Self-Administered Comorbidity Questionnaire (SCQ), a questionnaire of a self-administered measure of comorbidity validated for clinical and health services research settings [10]. This questionnaire is particularly useful because of its understandability and shortness giving us the possibility to assess in a concise and comprehensive manner the comorbidities of our study population as summarized in Table S2 (see supporting information). The questionnaire includes 12 medical conditions; through three “yes” or “no” questions, the score ranges between 0 (no pathology) and 3 (condition limiting the daily activity). To minimize an interviewer bias, the telephone survey was performed by the same investigator, following a standardized approach, ensuring the total anonymity to the other investigators who would subsequently conduct the statistical analysis of the results. This score allowed us to identify any possible significant comorbidity developed, along our population, between the surgery and the survey [10].

### Statistical analysis

A linear regression was trained with stepwise model selection by Akaike information criterion (AIC) using the caret package in R statistical software. A 10-fold cross-validation was used to estimate the residual mean squared error (RMSE). A two-tailed *t* test was used to estimate the significance of each variable. *P*-values < 0.05 were considered statistically significant.

Continuous data were expressed as the mean ± standard deviation or median and range as indicated. Correlation between GIQLI and other variables was assessed with Pearson’s coefficients.

## Results

**Fig. 1:**
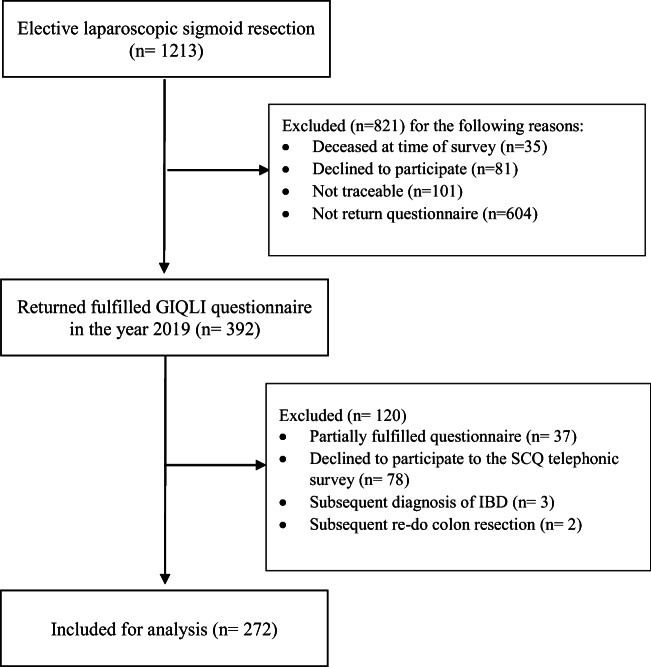
Flow chart of study design for gastrointestinal QoL analysis. GIQLI, gastrointestinal quality of life index; SCQ, self-administered comorbidity; IBD, inflammatory bowel disease

During the study period, 392 of 1213 patients undergoing to elective laparoscopic sigmoid resection for diverticular disease were enrolled in the study. Among these 392 patients, 277 filled the survey out correctly and answered to our subsequent SQC survey. After a stratification of different developed comorbidities, five patients were excluded from the final analysis: three due to their subsequent diagnosis of inflammatory bowel disease and two because they underwent additional abdominal surgery as described in the Fig. 1.

A baseline comparison between the 705 hypothetic eligible patients and the final 272 patients considered is summarized in Table S3 (see supporting information).

All demographic data at time of surgery, the pre-operative and intraoperative variables, as well as the postoperative morbidity of the eligible and included patients are listed in Table 1. The mean GIQLI of all the 272 patients was 116.39±18.25, while the mean follow-up time was 90.4 ± 33.65 months.

**Table 1:** All data of 392 qualified and 272 included patients that underwent to elective laparoscopic sigmoid resection for diverticular disease participated to gastrointestinal quality of life index (GIQLI) assessment and selected after attending to a Self-Administered Comorbidity Questionnaire (SCQ), where not otherwise indicated data are shown as numbers (n) and percentage (%)

	2004–2017	2004–2017
Number	392	272
Sex male/female	148 (37.7%)/244 (62.3%)	104 (38.2%)/168 (61.8%)
Age, mean ± standard deviation years	61.82 ± SD 10.22	62.30 ± SD 9.74
Pre-OP comorbidities	131 (33.4%)	87 (31.9%)
Immunosuppression	1	1
Diabetes mellitus	21	13
Coronary disease	29	17
Hypertension	108	72
History previous operations	172 (43.9%)	116 (42.6%)
Recurrent diverticulitis	344 (87.8%)	237 (87.1%)
Recurrent diverticulitis with covered perforation	34 (8.7%)	27 (9.9%)
Diverticular disease with enterovaginal fistula	1 (0.2%)	0
Diverticular disease with enterovesical fistula	2 (0.5%)	2 (0.8%)
Stenosing diverticular disease	11 (2.8%)	6 (2.2%)
Conversion laparotomy	27 (6.9%)	20 (7.3%)
Use of drains	215 (54.8%)	145 (53.3%)
IMA preserved	265 (67.6%)	201 (73.9%)
IMA resected	127 (32.4%)	71 (26.1%)
Anastomosis S-E	324 (82.6%)	219 (80.5%)
Anastomosis E-E	32 (8.2%)	24 (8.8%)
Anastomosis S-S	29 (7.4%)	22 (8.1%)
Anastomosis not applicable	7 (1.8%)	7 (2.6%)
Complications	69 (17.6%)	40 (14.7%)

**Fig. 2:**
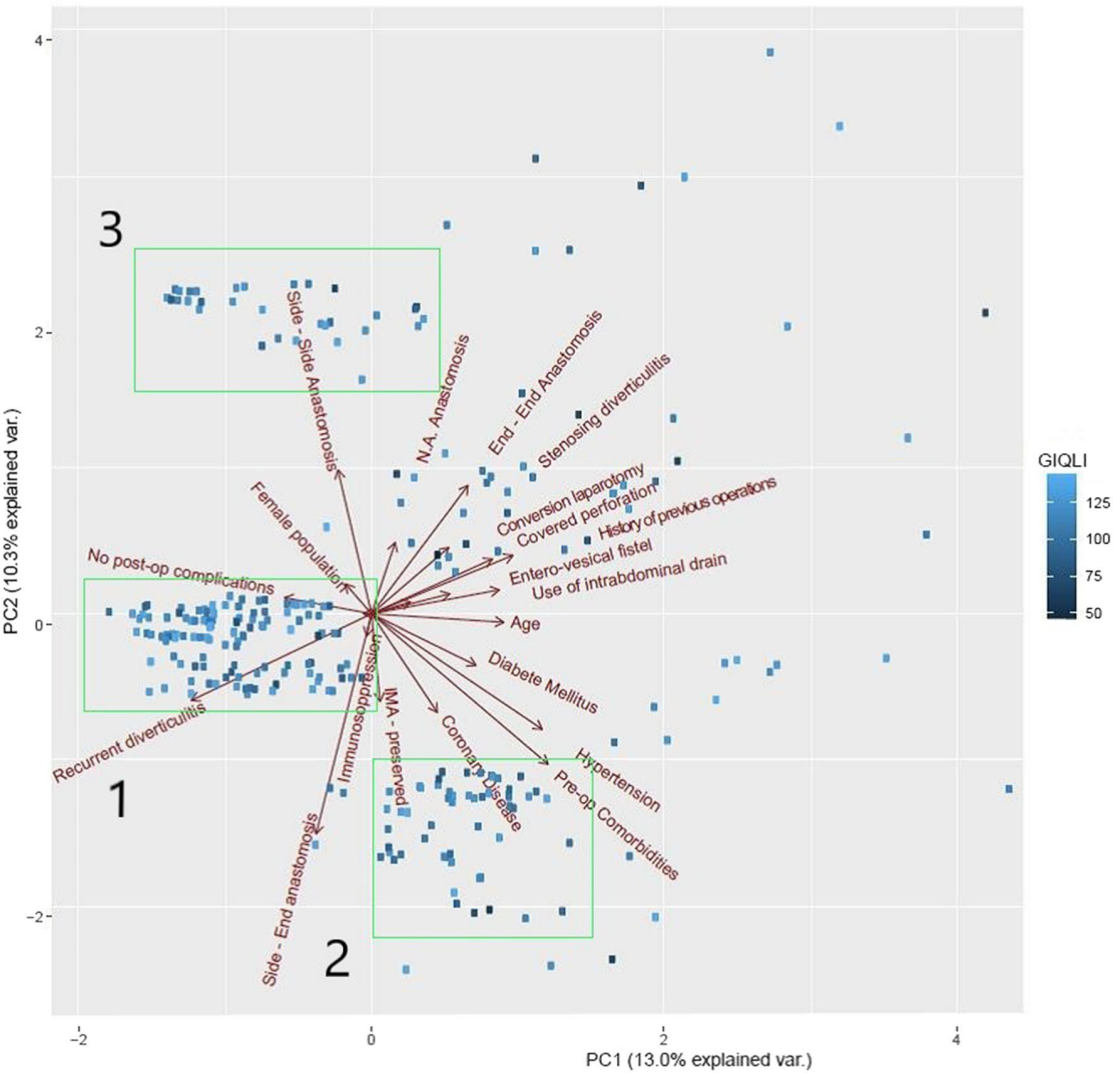
Principal component analysis (PCA) of 272 analyzed patients. Each patient is identified using blue squares, and the intensity of the color represents the GIQLI score. Red arrows represent the degree and direction of correlation of each perioperative variable with the principal components. PC1 represents the first principal direction along which the samples show the largest variation. PC2 represents the second most important direction. We have highlighted with green rectangles three different groups of patients that share similar perioperative conditions

The principal component analysis (PCA) of this cohort clustered patients in three main groups with similar characteristics, showing the relationship between these variables and the GIQLI as visualized in Fig. 2**.**

According to the linear regression model with stepwise selection, the gender, the time frame between the surgical procedure and our survey, and a pre-operative cardiovascular disease represent the conditions to better predict a decreased GIQLI (coefficient −2.369e-05, 1.355e-01, and −1.413e-01, respectively).

The female population (168 patients) showed a lower GIQLI compared to male (104 patients; 112.85 ±18.79 vs 122.11±15.81, *p*<0.001).

In addition, the comparison between both groups did not show any difference in terms of demographic and perioperative data, except for a predominance of history of previous operations in women and an intraoperative performed side-to-side anastomosis (51.7% vs 27.9%, *p*<0.001 and 11.3% vs 2.8%, *p*=0.012, respectively) as summarized in the Table 2. Nevertheless, the analysis of female sub-populations made by women with or without history of previous surgery confirms lower gastrointestinal quality of life index compared to male (87 patients; 111.89±19.62 vs 122.11±15.81, *p*<0.001, and 81 patients; 113.88 ±17.92 vs 122.11±15.81, *p*<0.001). In addition, the assessment of further actual comorbidities trough the telephonic survey of the Self-Administered Comorbidity Questionnaire (SCQ) showed no significant differences in the incidence of comorbidities along both groups (women: 83.3% vs men: 74.1%, *p*=0.063) excluding the prevalence of kidney disease in female population compared to the male one (*p*=0.042) (Table 2). A subsequent cross-comparison between all 5 different domains of the survey along both groups showed a significant worse GIQLI score in women compared to men, particularly among the subdomain core symptoms and disease-specific and psychological items as summarized in Table 3.

**Table 2:** Comparison of all the demographic data along the female and male population, including the Self-Administered Comorbidity Questionnaire (SQC) results

	Women	Men	*P* value
Number	168	104	
Age, years average ± SD	63.04±9.75	61.11±9.64	0.055
Time to operation, average months ±SD	90.36±34.67	90.46±31.12	0.490
Pre-OP comorbidities	47 (28%)	40 (38.4%)	0.082
Immunosuppression	0	1 (0.9%)	1
Diabetes mellitus	6 (3.6%)	7 (6.7%)	0.253
Coronary disease	11 (6.5%)	6 (5.8%)	1
Hypertension	41 (24.4%)	31 (29.8%)	0.326
History previous operations	87 (51.7%)	29 (27.9%)	**<0.001**
Recurrent diverticulitis	150 (89.2%)	87 (83.6%)	0.195
Recurrent diverticulitis with covered perforation	13 (7.7%)	14 (13.6%)	0.290
Diverticular disease with enterovaginal fistula	0	0	n.a.
Diverticular disease with enterovesical fistula	2 (1.2%)	0	0.525
Stenosing diverticular disease	3 (1.9%)	3 (2.8%)	0.677
Conversion laparotomy	12 (7.1%)	8 (7.7%)	1
Use of drains	89 (52.9%)	56 (53.8%)	0.901
IMA preserved	120 (71.4%)	81 (77.9%)	0.258
IMA resected	48 (28.6%)	23 (22.1%)	
Anastomosis S-E	132 (78.6%)	87 (83.6%)	0.346
Anastomosis E-E	12 (7.1%)	12 (11.7%)	0.271
Anastomosis S-S	19 (11.3%)	3 (2.8%)	**0.012**
Anastomosis not applicable	5 (3%)	2 (1.9%)	0.711
Complications	25 (14.9%)	15 (14.4%)	1
SQC scores*			
Number of patients (%)	140 (83.3%)	77 (74.1%)	0.063
Comorbidities, mean score			
Heart disease	0.27	0.38	0.116
Blood pressure	0.86	0.88	0.412
Lung disease	0.15	0.1	0.169
Diabetes	0.06	0.13	0.072
Ulcer/stomach disease	0.32	0.22	0.132
Kidney disease	0.05	0	**0.042**
Liver disease	0.01	0	0.216
Anemia other blood disease	0.08	0.12	0.274
Cancer	0.04	0.02	0.235
Depression	0.12	0.06	0.125
Degenerative arthritis	0.15	0.1	0.169
Back pain	0.14	0.07	0.085
Rheumatoid arthritis	0.08	0.04	0.158
Others	0.74	0.64	0.069

Significant differences are highlighted in bold

*SQC score ranges between 0 (no pathology) and 3 (condition limiting the daily activity)

**Table 3:** Sub-analysis of all the 5 different GIQLI domains and relative 36 specific questions applied to the female (*n*=168) and male (*n*=104) population

	Women	Men	*P* value
Core symptoms			
Abdominal pain	2.89±1.07	3.44±0.91	**<0.001**
Feeling of abdominal fullness	2.78±1.08	3.31±0.84	**<0.001**
Abdominal bloating (too much gas)	2.40±1.13	2.92±0.99	**<0.001**
Trouble with flatulence	2.48±1.12	2.93±1.01	**<0.001**
Trouble with burping or belching	3.23±0.97	3.28±0.92	0.356
Trouble with gurgling abdominal noises	3.07±0.93	3.27±0.77	**0.036**
Trouble with bowel frequency	2.84±10.9	3.34±0.95	**<0.001**
Enjoyed eating	3.40±0.84	3.48±0.82	0.213
Need for restricted eating	3.01±0.94	3.43±0.87	**<0.001**
Trouble with fatigue	2.53±0.92	2.85±0.94	**0.003**
Total score	28.36±6.80	32.18±5.71	**<0.001**
Psychological items			
Coping with every day stress	3.00±0.81	3.18±0.80	**0.036**
Sadness about illness	3.50±0.84	3.69±0.64	**0.024**
Nervousness or anxious about illness	3.50±0.74	3.68±0.66	**0.022**
Happiness with life in general	3.28±0.69	3.29±0.97	0.474
Frustration about illness	3.48±0.83	3.71±0.59	**0.006**
Total score	16.73±3.03	17.52±2.56	**0.013**
Physical items			
Feeling unwell	2.89±0.89	3.23±0.85	**<0.001**
Wake-up at night	1.54±1.50	2.28±1.44	**<0.001**
Trouble with changes in appearance	3.49±0.82	3.69±0.75	**0.021**
Loss of physical strength	3.27±0.90	3.38±0.93	0.151
Loss of endurance through illness	3.32±0.86	3.38±0.93	0.279
Feeling unfit	3.19±1.00	3.30±0.96	0.175
Total score	17.37±4.37	19.12±4.44	**<0.001**
Social items			
Coping with daily activities	3.73±0.71	3.87±0.34	**0.037**
Taking part in leisure activities	3.34±1.15	3.41±0.84	0.280
Bothered by medical treatment	3.72±0.64	3.76±0.59	0.286
Trouble of personal relationship	3.65±0.69	3.75±0.63	0.123
Sexual life impairment	3.53±0.95	3.21±1.17	**0.007**
Total score	17.18±3.66	17.79±2.58	0.068
Disease-specific items			
Regurgitation	3.56±0.81	3.60±0.78	0.356
Trouble with slow speed of eating	3.73±0.63	3.80±0.56	0.180
Trouble with dysphagia	3.74±0.59	3.78±0.64	0.323
Trouble with bowel urgency	2.71±1.02	3.17±0.92	**<0.001**
Trouble with diarrhea	2.99±0.97	3.34±0.85	**0.001**
Trouble with constipation	2.87±1.13	3.37±0.86	**<0.001**
Trouble with nausea	3.54±0.72	3.72±0.61	**0.017**
Trouble with blood in stool	3.94±0.28	3.86±0.45	**0.042**
Trouble with heartburn	3.22±1.02	3.29±0.92	0.280
Trouble with incontinence	3.33±0.94	3.65±0.68	**0.001**
Total score	33.41±5.02	35.50±4.29	**<0.001**

Significant differences are highlighted in bold

Moreover, no statistical difference in terms of GIQLI score has been noticed among 40 patients (14.7%) that developed postoperative complications compared to those with an uneventful postoperative course (232 patients; 112.98 ± 23.83 vs 116.97 ± 17.10, *p*=0.100).

Patients with a pre-operative cardiovascular disease (17 patients) had as well lower GIQLI compared to others (255 patients; 106.65 ±22.58 vs 117.08±17.66, *p*=0.010).

Finally, the GIQLI score improves progressively over the time. In fact, patients that underwent sigmoid resection more than 5 years before follow-up (209 patients) had a better GIQLI compared to the patients that underwent surgery less than 5 years ago (63 patients; 117.71±17.63 vs 111.98±19.65, *p*=0.014). More in detail, a selected comparison between patients operated within 5 years (63 patients) vs patients operated between 6 and 9 years ago (137 patients) or vs patients operated more than 10 years ago (72 patients) showed always a worse GIQLI in the first 5 years after surgery (111.98±19.65 vs 117.31±18.20, *p*=0.031 and 111.98±19.65 vs 118.49±16.60, *p*=0.019, respectively). On the contrary, no difference has been reported among patients operated between 6 and 9 years ago compared to those operated more than 10 years ago (117.31±18.20 vs 118.49±16.60, *p*=0.323).

Finally, the vascular approach to the IMA (central vs peripheral ligation) did not have any impact on the postoperative GIQLI (116.38±18.19 vs 116.39±18.53, *p*=0.498).

## Discussion

The current study investigated the long-term outcome of gastrointestinal function after elective laparoscopic sigmoid resection for diverticular disease in 272 patients. It demonstrated that female gender and the presence of pre-operative cardiovascular disease were predictive for a decreased postoperative GIQLI. Furthermore, a time lag below 5 years after surgery was associated with a lower gastrointestinal functioning compared to a longer follow-up. On the other hand, central dissection of the IMA using a high tie versus peripheral mesenteric dissection did not influence the long-term GIQLI.

In the last years, the guidelines on the treatment of diverticulitis have changed their recommendations, and the role of surgery has now evolved to a tailored approach focusing on the well-described improvement of gastrointestinal symptoms [11–14]. Despite this change in daily practice, to date few studies have deeply analyzed whether any patients or surgery-related variables could estimate the postoperative GIQLI. The knowledge of risk factors for poor gastrointestinal functioning after surgery could be thus of importance in the decision-making for elective surgery in chronic diverticular disease.

Forgione et al. compared the pre-operative and postoperative GIQLI in a small group of patients showing an increase of 10 points in patients undergoing to sigmoid resection. With a mean GIQLI of 111.5±20.4, 12 months after surgery, the score was in the same range as in the current study. The study by Forgione et al. confirmed the benefit of a surgical intervention, most of all in the improvement of disease-specific subdomain, but, unfortunately, the authors did not investigate any predictive variable responsible for this positive result [3, 5].

Through an exploratory principal component analysis, we have identified three main clusters of patients with similar characteristics. The cluster with the largest number of patients is mainly composed by young patients, with low frequency of pre-operative comorbidities that underwent surgery for recurrent diverticulitis. Those patients did not present postoperative morbidity. The second most populous cluster was represented by older patients, with pre-operative risk factors, who have benefited from a resection with preservation of the IMA. Finally, the third cluster is represented by the more complex cases, where the indication for surgery was due to a diagnosis of covered perforation or stenosis requiring, in the most of cases, a side-to-side anastomosis or temporary stoma.

This stratification of the cohort allowed confirming the heterogeneity of our population, showing that the QoL results were equally distributed in all three main clusters, thus excluding any possible selection bias in our further analysis.

A following linear regression model with stepwise selection allowed identifying the best predictive model, including the most influent variables, to achieve a good estimation of postoperative GIQLI. Interestingly, we noted that patients with previous cardiovascular disease and, most important, women had a significant worse postoperative GIQLI. By comparing in detail each answer of the assessment, we found that in 4 out of 5 different domains investigated, women consistently had a lower QoL compared to men, especially considering the core symptoms, the disease-specific and the physical and psychological items. This result seems to be independent of the higher rate of previous surgery in women. The analysis of sub-populations made by women with or without history of previous surgery confirmed lower GIQLI compared to male. The most significant differences indicate that in particular, abdominal pain, abdominal bloating with flatulence, and fecal urgency are the most crucial parameters negatively influencing the QoL. In literature, this aspect has been rarely investigated. Levack et al. retrospectively confirmed the results of the current analysis showing in his logistic regression analysis that high rates of fecal incontinence, fecal urgency, and also incomplete emptying after the sigmoid resection were predicted by female sex [8].

As already described in other studies, we also confirmed the tendency that over the time there is an improvement in the GIQLI [3, 5, 6]. In particular, within 5 years of surgery, the mean GIQLI was lower when compared with patients undergoing surgery more than 5 years ago. According to consistent literature, the general improvement over time of the GIQLI is probably due to biologic compensating mechanisms or, maybe together, to a psychological patient’s adaption to the surgical procedure and its postoperative outcomes [15, 16].

Interestingly, in the current analysis, central dissection of the IMA or peripheral mesenteric dissection did not influence the long-term GIQLI. Actually, the Italian Society of Colon and Rectal Surgery as well as the German Society of Colorectal disease recommend that the central ligation of IMA should be reserved in cases of suspected malignant disease or when the achieved colon mobilization is not enough to have a tension-free anastomosis [17, 18]. In the literature, the influence of a high tie dissection of the IMA on QoL was mostly studied in colorectal surgery for cancer [19–22]. The hypothesis by which the oncological resection could influence the postoperative QoL is based on anatomical reasons. The sympathetic nervous system originating from the inferior mesenteric plexus and the parasympathetic nervous system originating from the pelvic plexus innervate the descending and sigmoid colon, and they could be damaged during the meso-rectum plane dissection or central ligation of the IMA [23, 24]. Moreover, a sacrifice of the IMA could result in ischemia with consequently higher short-term complications or later anastomosis stenosis, and these scenarios could also be reflected in persisting or new onset of gastrointestinal symptoms [2, 4, 7, 10]. Unfortunately, few authors have evaluated the same risk in patients undergoing sigmoid resection for diverticular disease. The available studies concerning preservation or resection of the IMA in diverticular disease showed contradictory results. While Masoni et al. and Dobrowolski et al. reported a lower incidence of defecation disorders, fecal incontinence, and a greater QoL score in patients undergoing to IMA-preserved resection, Mari et al. demonstrated no differences between these different vascular approaches at 1 and 9 months after surgery [25–27]. Finally, our study, with a mean follow-up time of 90.4 ± 33.65 months, adds important evidence to the literature that the type of IMA ligation (central vs peripheral) does not represent a predictive estimator of the GIQLI.

The present study is inherently limited by its retrospective nature and a unique time-point assessment. First, the high drop-off rate, as described in table S3, represents an important limitation to notice. Secondly, the follow-up was assessed by questionnaires and not through a face-to-face interview with, if necessary, a clinical examination, influencing the quality of data collected. Moreover, according to the study design and the long follow-up, the lack of pre-operative GIQLI critically mitigates our conclusion. Keeping this weakness in mind, the clinical relevance of the results must be interpreted with caution. In particular, even if the analysis of female sub-populations, with or without previous history of surgery, consistently present a lower GIQLI compared to male, this difference needs to be carefully interpreted before applying in the decision-making process. In fact, a single time-point used to assess the postoperative gastrointestinal function without pre-operative time-point cannot deeply investigate this factor and its liability along the GIQLI score.

Finally, developing a postoperative complication does not seem to decrease the gastrointestinal quality of life, but this result must be interpreted with caution due to the lack of more specific data.

For all these reasons, we are considering applying our predictive model in a prospective study to verify the liability and the power of our results. Interestingly a protocol for a similar prospective, multicenter, and trans-national observational study has been recently published; the authors aim at identifying predictors of a postoperative change in quality of life in patients, comparing different surgical approaches [28].

## Conclusion

In conclusion, this observational study with a long-term follow-up of patients undergoing elective laparoscopic sigmoid resection for diverticular disease identified possible predictors of a decreased gastrointestinal QoL. Interestingly, female gender and the presence of cardiovascular disease seem to be the most predictive variables for poor postoperative QoL, while patients’ estimation of gastrointestinal functioning seems to improve over the time. However, current recommendation of different national surgical societies to preserve the IMA in order to maintain gastrointestinal function was not confirmed by the findings in the present trial. The preservation or resection of the IMA had no influence on the GIQLI in this study.

## Supplementary Information

ESM 1 (DOCX 21 kb)

## References

[1] Schilling PL, Dimick JB, Birkmeyer JD (2008). Prioritizing quality improvement in general surgery. J Am Coll Surg.

[2] Lin M, Raman SR (2018). Evaluation of quality of life and surgical outcomes for treatment of diverticular disease. Clin Colon Rectal Surg.

[3] Forgione A, Guraya SY (2016). Elective colonic resection after acute diverticulitis improves quality of life, intestinal symptoms and functional outcome: experts’ perspectives and review of literature. Updat Surg.

[4] Regenbogen SE, Hardiman KM, Hendren S, Morris AM (2014). Surgery for diverticulitis in the 21st century: a systematic review. JAMA Surg.

[5] Forgione A, Leroy J, Cahill RA, Bailey C, Simone M, Mutter D, Marescaux J (2009). Prospective evaluation of functional outcome after laparoscopic sigmoid colectomy. Ann Surg.

[6] Polese L, Bressan A, Savarino E, Vecchiato M, Turoldo A, Frigo A, Sturniolo GC, de Manzini N, Petri R, Merigliano S (2018). Quality of life after laparoscopic sigmoid resection for uncomplicated diverticular disease. Int J Color Dis.

[7] Egger B, Peter MK, Candinas D (2008). Persistent symptoms after elective sigmoid resection for diverticulitis. Dis Colon Rectum.

[8] Levack MM, Savitt LR, Berger DL, Shellito PC, Hodin RA, Rattner DW, Goldberg SM, Bordeianou L (2012). Sigmoidectomy syndrome? Patients’ perspectives on the functional outcomes following surgery for diverticulitis. Dis Colon Rectum.

[9] Eypasch E, Williams JI, Wood-Dauphinee S, Ure BM, Schmülling C, Neugebauer E, Troidl H (1995). Gastrointestinal quality of life index: development, validation and application of a new instrument. Br J Surg.

[10] Sangha O, Stucki G, Liang MH, Fossel AH, Katz JN (2003). The self-administered comorbidity questionnaire: a new method to assess comorbidity for clinical and health services research. Arthritis Rheum.

[11] Sartelli M, Weber DG, Kluger Y, Ansaloni L, Coccolini F, Abu-Zidan F, Augustin G, Ben-Ishay O, Biffl WL, Bouliaris K, Catena R, Ceresoli M, Chiara O, Chiarugi M, Coimbra R, Cortese F, Cui Y, Damaskos D, de’ Angelis GL, Delibegovic S, Demetrashvili Z, de Simone B, di Marzo F, di Saverio S, Duane TM, Faro MP, Fraga GP, Gkiokas G, Gomes CA, Hardcastle TC, Hecker A, Karamarkovic A, Kashuk J, Khokha V, Kirkpatrick AW, Kok KYY, Inaba K, Isik A, Labricciosa FM, Latifi R, Leppäniemi A, Litvin A, Mazuski JE, Maier RV, Marwah S, McFarlane M, Moore EE, Moore FA, Negoi I, Pagani L, Rasa K, Rubio-Perez I, Sakakushev B, Sato N, Sganga G, Siquini W, Tarasconi A, Tolonen M, Ulrych J, Zachariah SK, Catena F (2020). update of the WSES guidelines for the management of acute colonic diverticulitis in the emergency setting. World J Emerg Surg. 2020.

[12] Floch MH, Longo WE (2016). United States guidelines for diverticulitis treatment. J Clin Gastroenterol.

[13] Siddiqui J, Zahid A, Hong J, Young CJ (2017). Colorectal surgeon consensus with diverticulitis clinical practice guidelines. World J Gastrointest Surg.

[14] Galata C, Lock JF, Reißfelder C, Germer CT (2020). Empfehlungen zur Therapie der Divertikelkrankheit [Recommendations for treatment of diverticular disease]. Chirurg..

[15] van de Wall BJM, Stam MAW, Draaisma WA, Stellato R, Bemelman WA, Boermeester MA, Broeders IAMJ, Belgers EJ, Toorenvliet BR, Prins HA, Consten ECJ, DIRECT trial collaborators (2017). Surgery versus conservative management for recurrent and ongoing left-sided diverticulitis (DIRECT trial): an open-label, multicentre, randomised controlled trial. Lancet Gastroenterol Hepatol.

[16] Patel SV, Hendren S, Zaborowski A, Winter D, for Members of the Evidence Based Reviews in Surgery group (2020). Evidence-based reviews in surgery long-term outcome of surgery versus conservative management for recurrent and ongoing complaints after an episode of diverticulitis: five-year follow-up results of a multicenter randomized controlled trial (DIRECT-Trial). Ann Surg.

[17] Binda GA, Cuomo R, Laghi A, Nascimbeni R, Serventi A, Bellini D, Gervaz P, Annibale B, Italian Society of Colon and Rectal Surgery (2015). Practice parameters for the treatment of colonic diverticular disease: Italian Society of Colon and Rectal Surgery (SICCR) guidelines. Tech Coloproctol.

[18] Leifeld L, Germer CT, Böhm S (2014). S2k-Leitlinie Divertikelkrankheit/Divertikulitis [S2k guidelines diverticular disease/diverticulitis]. Z Gastroenterol.

[19] Walming S, Asplund D, Bock D et al (2020) Quality of life in patients with resectable rectal cancer during the first 24 months following diagnosis [published online ahead of print, 2020 Sep 1]. Color Dis. 10.1111/codi.15343 10.1111/codi.15343 PMC7821207 32871612

[20] Zhao GH, Deng L, Ye DM (2020). Efficacy and safety of wait and see strategy versus radical surgery and local excision for rectal cancer with cCR response after neoadjuvant chemoradiotherapy: a meta-analysis. World J Surg Oncol.

[21] Koplin G, Müller V, Heise G, Pratschke J, Schwenk W, Haase O (2016). Effects of psychological interventions and patients’ affect on short-term quality of life in patients undergoing colorectal surgery. Cancer Med.

[22] Boullenois H, Lefevre JH, Creavin B, Calmels M, Voron T, Debove C, Chafai N, Parc Y (2019). What is the functional result of a delayed coloanal anastomosis in redo rectal surgery?. ANZ J Surg.

[23] Feher J. 8.3 - Intestinal and colonic chemoreception and motility. In: Feher J, ed. Quantitative Human Physiology (Second Edition). Boston: Academic Press; 2017: 796-809.

[24] Moriya Y (2006). Function preservation in rectal cancer surgery. Int J Clin Oncol.

[25] Dobrowolski S, Hać S, Kobiela J, śledziński Z (2009). Should we preserve the inferior mesenteric artery during sigmoid colectomy?. Neurogastroenterol Motil.

[26] Masoni L, Mari FS, Nigri G, Favi F, Gasparrini M, Dall’Oglio A, Pindozzi F, Pancaldi A, Brescia A (2013). Preservation of the inferior mesenteric artery via laparoscopic sigmoid colectomy performed for diverticular disease: real benefit or technical challenge: a randomized controlled clinical trial. Surg Endosc.

[27] Mari G, Crippa J, Costanzi A, Mazzola M, Magistro C, Ferrari G, Maggioni D (2017). Genito-urinary function and quality of life after elective totally laparoscopic sigmoidectomy after at least one episode of complicated diverticular disease according to two different vascular approaches: the IMA low ligation or the IMA preservation. Chirurgia (Bucur).

[28] Sohn M, Agha A, Iesalnieks I (2020). PREDICtors for health-related quality of life after elective sigmoidectomy for DIVerticular disease: the PREDIC-DIV study protocol of a prospective multicentric transnational observational study. BMJ Open.

