# Supplementary material for: Predictive model estimating the decrease of postoperative gastrointestinal quality of life index (GIQLI) in patients after elective laparoscopic sigmoid resection for diverticular disease
Source: Langenbecks Arch Surg. 2021 May 25;406(5):1571–80. doi: 10.1007/s00423-021-02186-w (PMC8370950; doi:10.1007/s00423-021-02186-w)
Supplement: Supplementary file 1 — (DOCX 21 kb) [file 423_2021_2186_MOESM1_ESM.docx]

**Supporting Information:**

**Table S1:** 36 item Gastrointestinal Quality of Life Index (GIQLI) divided according to the 5 different subdomains

| **Gastrointestinal Quality of Life Index**  **(GIQLI)** | **All of the time** | **Most of the time** | **Some of the time** | **A little of the time** | **Never** |
| --- | --- | --- | --- | --- | --- |
| **Core Symptoms** |  |  |  |  |  |
| Abdominal pain | 0 | 1 | 2 | 3 | 4 |
| Feeling of abdominal fullness | 0 | 1 | 2 | 3 | 4 |
| Abdominal bloating (too much gas) | 0 | 1 | 2 | 3 | 4 |
| Trouble with flatulence | 0 | 1 | 2 | 3 | 4 |
| Trouble with burping or belching | 0 | 1 | 2 | 3 | 4 |
| Trouble with gurgling abdominal noises | 0 | 1 | 2 | 3 | 4 |
| Trouble with bowel frequency | 0 | 1 | 2 | 3 | 4 |
| Enjoyed eating | 0 | 1 | 2 | 3 | 4 |
| Need for restricted eating | 0 | 1 | 2 | 3 | 4 |
| Trouble with fatigue | 0 | 1 | 2 | 3 | 4 |
|  |  |  |  |  |  |
| **Psychological items** |  |  |  |  |  |
| Coping with every day stress | 0 | 1 | 2 | 3 | 4 |
| Sadness about illness | 0 | 1 | 2 | 3 | 4 |
| Nervousness or anxious about illness | 0 | 1 | 2 | 3 | 4 |
| Happiness with life in general | 0 | 1 | 2 | 3 | 4 |
| Frustration about illness | 0 | 1 | 2 | 3 | 4 |
|  |  |  |  |  |  |
| **Physical items** |  |  |  |  |  |
| Feeling unwell | 0 | 1 | 2 | 3 | 4 |
| Wake-up at night | 0 | 1 | 2 | 3 | 4 |
| Trouble with changes in appearance | 0 | 1 | 2 | 3 | 4 |
| Loss of physical strength | 0 | 1 | 2 | 3 | 4 |
| Loss of endurance through illness | 0 | 1 | 2 | 3 | 4 |
| Feeling unfit | 0 | 1 | 2 | 3 | 4 |
|  |  |  |  |  |  |
| **Social items** |  |  |  |  |  |
| Coping with daily activities | 0 | 1 | 2 | 3 | 4 |
| Taking part in leisure activities | 0 | 1 | 2 | 3 | 4 |
| Bothered by medical treatment | 0 | 1 | 2 | 3 | 4 |
| Trouble of personal relationship | 0 | 1 | 2 | 3 | 4 |
| Sexual life impairment | 0 | 1 | 2 | 3 | 4 |
|  |  |  |  |  |  |
| **Disease-specific items** |  |  |  |  |  |
| Regurgitation | 0 | 1 | 2 | 3 | 4 |
| Trouble with slow speed of eating | 0 | 1 | 2 | 3 | 4 |
| Trouble with dysphagia | 0 | 1 | 2 | 3 | 4 |
| Trouble with bowel urgency | 0 | 1 | 2 | 3 | 4 |
| Trouble with diarrhea | 0 | 1 | 2 | 3 | 4 |
| Trouble with constipation | 0 | 1 | 2 | 3 | 4 |
| Trouble with nausea | 0 | 1 | 2 | 3 | 4 |
| Trouble with blood in stool | 0 | 1 | 2 | 3 | 4 |
| Trouble with heartburn | 0 | 1 | 2 | 3 | 4 |
| Trouble with incontinence | 0 | 1 | 2 | 3 | 4 |

**Table S2:** Self-Administered Comorbidity Questionnaire (SCQ)

|  | **Do you have the problem ?** | **Do you receive treatment for it ?** | **Does it limit your activities?** |
| --- | --- | --- | --- |
|  | **No (0) / Yes (1)** | **No (0) / Yes (1)** | **No (0) / Yes (1)** |
| **Co-Morbidities** |  |  |  |
| **heart disease** | **0 / 1** | **0 / 1** | **0 / 1** |
| **blood pressure** | **0 / 1** | **0 / 1** | **0 / 1** |
| **lung disease** | **0 / 1** | **0 / 1** | **0 / 1** |
| **diabetes** | **0 / 1** | **0 / 1** | **0 / 1** |
| **ulcer or stomach disease** | **0 / 1** | **0 / 1** | **0 / 1** |
| **kidney disease** | **0 / 1** | **0 / 1** | **0 / 1** |
| **liver disease** | **0 / 1** | **0 / 1** | **0 / 1** |
| **anemia other blood disease** | **0 / 1** | **0 / 1** | **0 / 1** |
| **cancer** | **0 / 1** | **0 / 1** | **0 / 1** |
| **depression** | **0 / 1** | **0 / 1** | **0 / 1** |
| **degenerative arthritis** | **0 / 1** | **0 / 1** | **0 / 1** |
| **back pain** | **0 / 1** | **0 / 1** | **0 / 1** |
| **rheumatoid arthritis** | **0 / 1** | **0 / 1** | **0 / 1** |
| **others** | **0 / 1** | **0 / 1** | **0 / 1** |

**Table S3:** Demographic data comparison between the eligible population and the final population analyzed GIQLI score. The eligible population (705 of 1213 patients; 58.1%) consisted of patients not traceable (101) and those that did not return the questionnaire (604).

|  | **Eligible population**  **(n=705)** | **Final population analyzed**  **(n=272)** |
| --- | --- | --- |
|  |  |  |
| **Age, mean ± standard deviation** | 63.01 ±10.94 | 62.30 ±9.74 |
| **Sex ratio male/female** | 260 (36.9%) / 455(63.1%) | 104 (38.2%) /168 (61.8%) |
| **Pre-OP comorbidities** | 247 (35%) | 87 (31.9%) |
| **Immunosuppression** | 7 | 1 |
| **Diabetes Mellitus** | 34 | 13 |
| **Coronary disease** | 55 | 17 |
| **Hypertension** | 189 | 72 |
| **History of previous operations** | 279 (39.6%) | 116 (42.6%) |
| **Indications for operative treatment** |  |  |
| **Recurrent diverticulitis** | 597 (84.7%) | 237 (87.1%) |
| **Recurrent diverticulitis with covered perforation** | 63 (8.9%) | 27 (9.9%) |
| **Diverticular disease with enterovaginal fistula** | 3 (0.4%) | 0 |
| **Diverticular disease with enterovesical fistula** | 7 (1%) | 2 (0.8%) |
| **Stenosing diverticular disease** | 35 (4.9%) | 6 (2.2%) |
| **Conversion laparotomy** | 62 (8.8%) | 20 (7.3%) |
| **Use of drains** | 359 (51%) | 145 (53.3%) |
| **IMA resected/preserved** | 178 (25.3%) /527 (74.7%) | 71 (26.1%) / 201 (73.9%) |
| **Kind of anastomosis** |  |  |
| **Anastomosis Side-End** | 569 (80.7%) | 219 (80.5%) |
| **Anastomosis End-End** | 68 (9.6%) | 24 (8.8%) |
| **Anastomosis Side-Side** | 61 (8.6%) | 22 (8.1%) |
| **Anastomosis not applicable** | 7 (1.1%) | 7 (2.6%) |
